# miR-449a causes Rb-dependent cell cycle arrest and senescence in prostate cancer cells

**DOI:** 10.18632/oncotarget.167

**Published:** 2010-09-13

**Authors:** Emily J. Noonan, Robert F. Place, Shashwati Basak, Deepa Pookot, Long-Cheng Li

**Affiliations:** ^1^Center for Molecular Biology in Medicine, Veterans Affairs Palo Alto Health Care System, Palo Alto, CA, USA; ^2^Department of Medicine, Stanford University School of Medicine, Stanford, CA, USA; ^3^Helen Diller Comprehensive Cancer Center, University of California, San Francisco, CA, USA; ^4^Department of Urology, University of California, San Francisco, CA, USA; ^5^Tacere Therapeutics Inc., San Jose, CA, USA

**Keywords:** Prostate cancer, miRNA, miR-449a, retinoblastoma, CCND1, HDAC1, p27, cell cycle, cellular senescence

## Abstract

MicroRNAs (miRNAs) are a class of small non-coding RNAs (ncRNAs) that regulate gene expression by repressing translation or triggering the degradation of complementary mRNA sequences. Certain miRNAs have been shown to function as integral components of the p53 and/or retinoblastoma (Rb) regulatory networks. As such, miRNA dysregulation can have a profound effect on cancer development. Previous studies have shown that miR-449a is down-regulated in human prostate cancer tissue and possesses potential tumor suppressor function. In the present study, we identify miR-449a-mediated growth arrest in prostate cancer cells is dependent on the Rb protein. We show that mutant Rb prostate cancer cells (DU-145) are resistant to cell cycle arrest and cellular senescence induced by miR-449a, while overexpression of wild-type Rb in DU-145 sublines (DU-1.1 and B5) restores miR-449a function. *In silico* analysis of 3'UTR regions reveal a putative miR-449a target site in the transcript of Cyclin D1 (CCND1); an oncogene involved in directly regulating Rb activity and cell cycle progression. Luciferase 3'UTR reporter constructs and inhibitory oligonucleotides confirm that Cyclin D1 is a direct downstream target of miR-449a. We also reveal that miR-449a suppresses Rb phosphorylation through the knockdown of Cyclin D1 and previously validated target HDAC1. By targeting genes involved in controlling Rb activity, miR-449a regulates growth and senescence in an Rb-dependent manner. These data indicate that miR-449a is a miRNA component of the Rb pathway and its tumor suppressor-like effects, in part, depends on Rb status in prostate cancer cells.

## INTRODUCTION

MicroRNAs (miRNAs) are a class of small non-coding RNAs (ncRNAs) that function as key regulators of gene expression. By targeting complementary sequences in gene transcripts, miRNAs inhibit translation or degrade mRNAs to silence gene expression [1, 2]. Based on this function, miRNAs have been implicated in a wide range of cellular processes including cellular growth, differentiation, and disease [3].

Cancer is the result of a complex multistep process that involves the accumulation of sequential alterations of several genes. Retinoblastoma (Rb) protein is frequently mutated or inactivated in a broad range of cancer types to promote cell proliferation and survival. In prostate cancer, Rb inactivation is equally crucial for malignant transformation, which has been reported to be absent in as much as 60% of all lesions [4-6]. Mutations or overexpression of genes involved in suppressing Rb activity through hyperphosphorylation are also important components in cancer progression. For instance, constitutive activation of Cyclin D1 (CCND1) has been shown to promote Rb phosphorylation and cellular growth in numerous cancers including prostate cancer [7-9].

Aberrant miRNA expression is also linked to a number of human cancers [10]. Based on downstream targets, miRNAs can function as tumor suppressors and/or oncogenes. For instance, miR-34a is a tumor suppressor miRNA directly regulated by p53 [11]. It has proven to be an integral component of the p53 pathway by suppressing cell growth through targeting various oncogenes (i.e. MYCN, c-Met, etc.) [12, 13]. As such, the tumor suppressor activity of p53 has been directly linked to the growth inhibitory effects of miR-34a [14, 15].

It has been shown that miR-449a is depleted in human prostate tumor tissue relative to patient-matched controls and possesses tumor suppressor-like function, in part, through targeting HDAC1 and activating p27 expression [16]. In the present study, we indicate that the growth inhibitory function of miR-449a is largely dependent on Rb status in prostate cancer cells. Mutant Rb renders DU-145 cells generally resistant to the growth inhibitory effects of miR-449a, while DU-145 sublines expressing wild-type Rb retain sensitivity to miR-449a. The dependency on Rb is likely facilitated through miR-449a targeting genes involved in regulating Rb activity. In support, we define Cyclin D1 as another direct target suppressed by miR-449a in prostate cancer cells. We further indicate that miR-449a suppresses Rb phosphorylation through specific knockdown of both Cyclin D1 and HDAC1. Our data implicates miR-449a as a key miRNA component of the Rb pathway that functions to regulate prostate cancer cell growth, in part, by controlling Rb activity.

## RESULTS

### DU-145 cells are resistant to miR-449a-induced growth arrest and senescence

**Figure 1:**
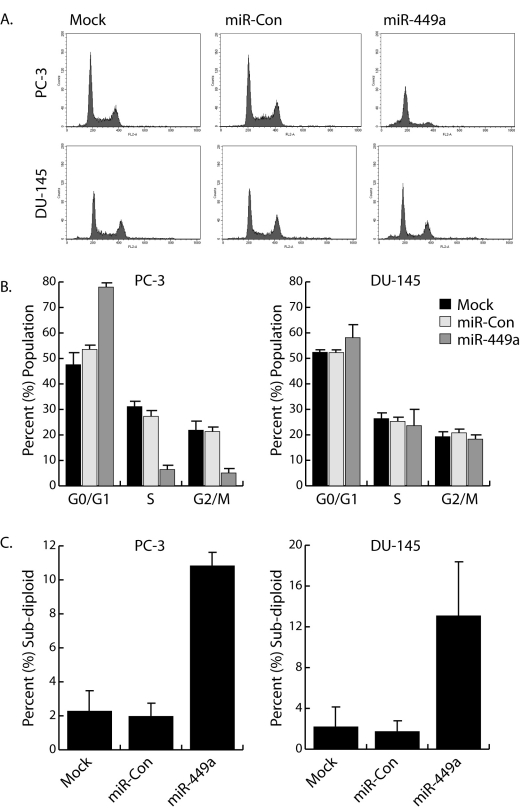
Differential effects of miR-449a on cell cycle distribution in PC-3 and DU-145 cells **A.** PC-3 and DU-145 cells were transfected with 50 nM concentrations of miR-Con or miR-449a for 72 hours. Mock samples were transfected in the absence of miRNA. Floating and attached cells were collected, stained with PI, and processed for analysis by flow cytometry to measure DNA content. Shown are examples of resulting FL2A histograms. **B.** Flow cytometry data was analyzed to determine cell cycle distribution (G0/G1, S, and G2/M) in the surviving cell populations. **C.** Percentages of sub-diploid/apoptotic cells were calculated from entire gated whole-cell populations.

MicroRNA-449a is depleted in human prostate tumors and functions as a potential tumor suppressor miRNA by inhibiting proliferation and promoting apoptosis [16]. In order to further examine the physiological effects of miR-449a on prostate cancer cell growth, we transfected PC-3 (prostate adenocarcinoma) and DU-145 (prostate carcinoma) cells with miR-449a and evaluated cell cycle distribution by flow cytometry. Cells were also transfected with a non-specific miRNA (miR-Con) to serve as a control. In PC-3 cells, miR-449a caused G0/G1 arrest as indicated by the increase in G0/G1 cell number and corresponding reductions in S and G2/M populations (Figure 1A-B). In contrast, cell cycle distribution in DU-145 cells was not significantly altered by miR-449a (Figure 1A-B). Further analysis of DNA fragmentation/apoptosis revealed a substantial increase in cells with sub-diploid DNA content; miR-449a caused ~11% and ~13% boost in PC-3 and DU-145 apoptotic cell populations, respectively (Figure 1C). Although sensitive to the apoptotic effects of miR-449a, DU-145 cells are resistant to the inhibitory function of miR-449a on cell cycle progression.

**Figure 2:**
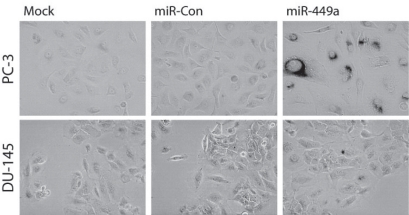
DU-145 cells are resistant to miR-449a-induced cellular senescence PC-3 and DU-145 cells were transfected with 50 nM concentrations of miR-Con or miR-449a for 72 hours. Cells were fixed in formaldehyde and stained for SA-β-gal activity overnight. Images were captured by phase contrast microscopy at 200X magnification. Dark perinuclear staining marks senescent cells.

All cancer cells must circumvent cellular senescence in order to become immortal. General dysregulation of the cell cycle and key regulatory genes (i.e. p21, p16, etc.) can effectively promote a senescent-like phenotype in cancer cells [17-20]. Detection of senescence associated β-galactosidase (SA-β-gal) activity at pH 6.0 is a known biomarker for cellular senescence [21]. To evaluate miR-449a-induced senescence in prostate cancer cell lines, we transfected PC-3 and DU-145 cells with miR-449a and stained for SA-β-gal activity. In a pattern similar to cell cycle analysis, PC-3 cells stained positive for SA-β-gal activity following miR-449a transfection, while staining in DU-145 cells was nearly undetectable in all treatments (Figure 2). These results further suggest that DU-145 cells are generally resistant to the growth arrest effects of miR-449a.

### Rb sensitizes DU-145 sublines to growth arrest by miR-449a

**Figure 3:**
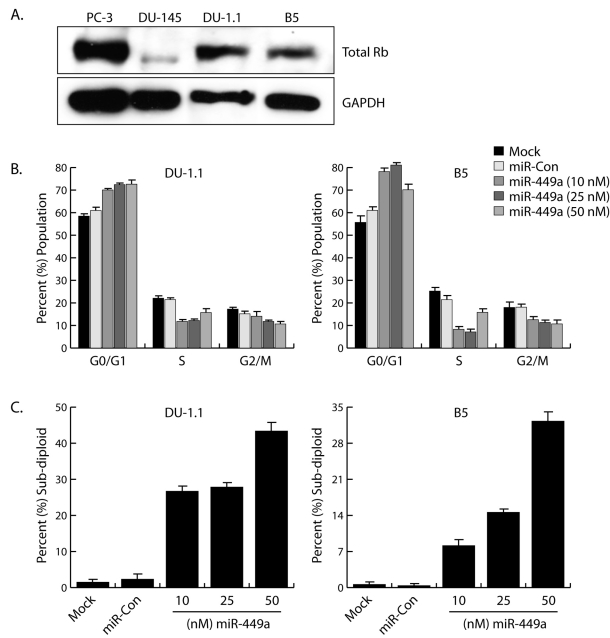
Wild-type Rb sensitizes DU-1.1 and B5 cells to miR-449a-mediate cell cycle arrest **A.** Protein extracts were prepared from PC-3, DU-145, DU-1.1, and B5 cell lines and resolved on SDS polyacrylamide gels. Total Rb and GAPDH were detected by immunoblot analysis in each cell line. GAPDH served as a loading control. Note DU-145 cells express low levels of a slightly truncated form of Rb. **B.** DU-1.1 and B5 cells were transfected with mock, miR-Con, or miR-449a for 72 hours as indicated. Floating and attached cells were collected, stained with PI, and processed for analysis by flow cytometry to measure DNA content. Data was analyzed to determine cell cycle distribution (G0/G1, S, and G2/M) in the surviving cell populations. Lower concentrations of miR-449a improved cell cycle analysis by increasing viable cell number. **C.** Percent sub-diploid/apoptotic cells were calculated from entire gated whole-cell populations.

Cellular senescence is primarily mediated by two proteins – p53 and retinoblastoma (Rb). Interestingly, neither PC-3 nor DU-145 cells express functional p53; however, wild-type Rb protein is expressed in PC-3 cells [22, 23]. DU-145 cells possess a loss-of-function mutation that yields a truncated Rb protein [23]. To determine if wild-type Rb can sensitize DU-145 cells to miR-449a, we obtained two DU-145 sublines that stably overexpress wild-type Rb from either a plasmid vector (DU-1.1) or retroviral construct (B5). As shown in Figure 3A, detection of Rb protein was validated by immunoblot analysis. Both DU-1.1 and B5 cells express wild-type Rb at levels comparable to PC-3 cells, while DU-145 cells express low-levels of a slightly truncated form of Rb. We subsequently transfected both DU-1.1 and B5 cells with miR-449a and examined cell cycle distribution by flow cytometry. As shown in Figure 3B, miR-449a caused G0/G1 arrest in DU-1.1 and B5 cells as indicated by the increase in G0/G1 cell number and concurrent declines in S and G2/M populations. Rb also enhanced the apoptotic effects of miR-449a; sub-diploid/apoptotic populations increased to as much as ~45% and ~30% in DU-1.1 and B5, respectively (Figure 3C). Because standard miR-449a transfection concentrations (50 nM) resulted in robust levels of cell death, DU-1.1 and B5 cells were also transfected at lower concentrations (10 and 25 nM) of miR-449a to increase viable cell number. Note improvement in cell quantity within the cell cycle fraction at lower concentrations of miR-449a in the FLA2 histograms (Supplementary Figure 1). 10 and 25 nM treatments reduced the apoptotic cell fractions and improved cell cycle analysis of DU-1.1 and B5 cells (Figure 3B-C). Collectively, these results indicate that wild-type Rb sensitizes DU-145 sublines to miR-449a-induced growth arrest, as well as further enhances the apoptotic effects of miR-449a.

**Figure 4:**
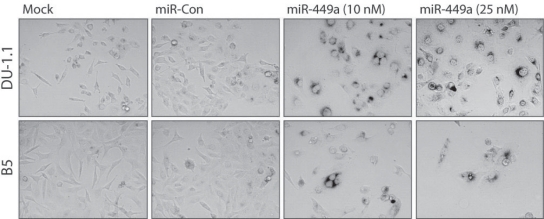
miR-449a triggers cellular senescence in DU-1.1 and B5 cells DU-1.1 and B5 cells were transfected with mock, miR-Con, or miR-449a for 72 hours. Transfection of miR-449a at 10 or 25 nM concentrations improved cell density for analysis. Cells were fixed in formaldehyde and stained for SA-β-gal activity overnight. Images were captured by phase contrast microscopy at 200X magnification. Dark perinuclear staining marks senescent cells.

To determine if DU-1.1 and B5 cells have also become sensitive to miR-449a-induced senescence, we transfected DU-145 sublines with miR-449a and stained for SA-β-gal activity. At 10 and 25 nM concentrations, DU-1.1 and B5 cells stained positive for SA-β-gal, while staining in mock and miR-Con treatments were nearly undetectable (Figure 4). This data indicates that miR-449a triggered a senescent-like phenotype in both DU-145 sublines.

DuPro (prostate adenocarcinoma) cells are also wild-type for Rb [22, 24]. Transfection of miR-449a promoted cell cycle arrest at G1/G0, increased sub-diploid/apoptotic populations, and caused SA-β-gal staining to indicate that DuPro cells are also sensitive to miR-449a (Supplementary Figure 2A-D). This data further supports that miR-449a induces growth arrest *via* an Rb-dependent mechanism in prostate cancer cells.

### miR-449a targets Cyclin D1

**Figure 5:**
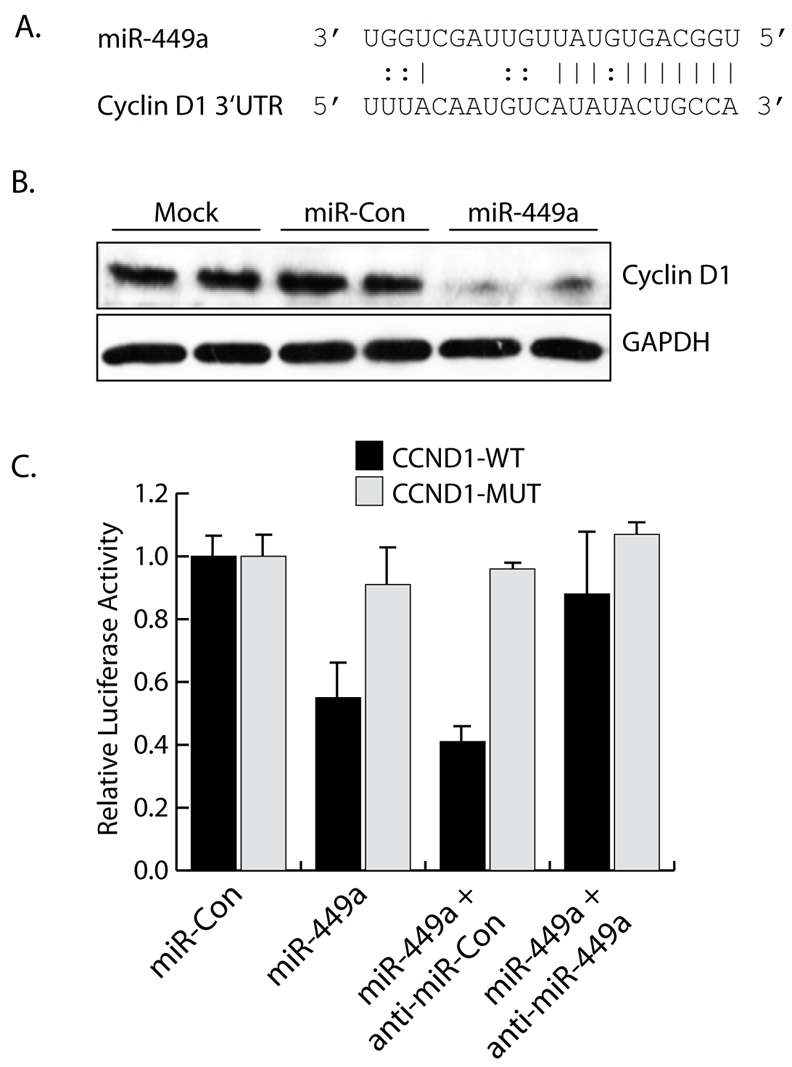
miR-449a targets Cyclin D1 **A.** The 3'UTR of the Cyclin D1 transcript contains a putative miR-449a target site. Indicated is complementary base-pairing, including G:U wobbles, between the mature miR-449a guide sequence and the Cyclin D1 target site. **B.** PC-3 cells were transfected with mock, miR-Con, or miR-449a for 72 hours as indicated. Cyclin D1 and GAPDH protein levels were evaluated by immunoblot analysis using protein-specific antibodies. GAPDH served as a loading control. **C.** Putative miR-449a target sequence from Cyclin D1 (CCND1-WT) was cloned into the 3'UTR of a luciferase reporter vector. A scrambled target site (CCND1-MUT) was also constructed as a control for sequence specificity. Constructs were co-transfected with a β-galactosidase expression vector and treated with miR-449a for 24 hrs. Luciferase activity was accessed and normalized to β-galactosidase activity. Cells were also co-treated with a miR-449a inhibitory oligonucleotide (anti-miR-449a) or a non-specific control (anti-miR-Con) to confirm reduction in luciferase activity was dependent on miR-449a sequence.

Because Rb is required, in part, for the tumor suppressor-like function of miR-449a in prostate cancer cells, miR-449a likely targets genes responsible for regulating Rb activity. Cyclin D1 functions in conjunction with CDK4/6 to directly regulate Rb phosphorylation and promote entry into S phase of the cell cycle [8]. As shown in Figure 5A, *in silico* analysis revealed a putative target site in the 3'UTR of the Cyclin D1 (CCND1) transcript. Because all modes of miRNA-mediated gene repression result in decreased target protein, we evaluated Cyclin D1 levels by immunblot analysis. As shown in Figure 5B, miR-449a significantly reduced Cyclin D1 protein levels in PC-3 cells. To confirm Cyclin D1 is a direct target of miR-449a, we cloned the putative target sequence into the 3'UTR of a luciferase reporter vector (CCND1-WT). A scrambled target site (CCND1-MUT) was also constructed as a control for sequence specificity. Co-transfection with miR-449a reduced the luciferase activity of CCND1-WT, whereas the Cyclin D1 mutant construct (CCND1-MUT) was protected from miR-449a-mediated repression (Figure 5C). We also co-treated cells with a complementary oligonucleotide (anti-miR-449a) designed to specifically bind and sequester miR-449a activity. Although transfection of a non-specific control oligonucleotide (anti-miR-Con) did not interfere with the miR-449a-mediated repression of CCND1-WT, anti-miR-449a blocked miR-449a function causing a rebound in CCND1-WT luciferase activity (Figure 5C). Taken together, this data indicates that the Cyclin D1 transcript is a direct target of miR-449a.

Conservation of miRNA and target site sequence across multiple species is considered supporting evidence for authentic miRNA-target interactions [25]. Interestingly, miR-449a has already been established as an evolutionary conserved miRNA [16]. We performed an additional *in silico* analysis on the Cyclin D1 3'UTR and identified the miR-449a target site as a highly-conserved sequence found in many vertebrates (i.e. human, horse, lizard, etc.) (Supplementary Figure 3). This highlights an evolutionary significance for the target site and corroborates the functional interaction between the Cyclin D1 transcript and miR-449a.

### miR-449a regulates Rb phosphorylation by targeting Cyclin D1 and HDAC1

**Figure 6:**
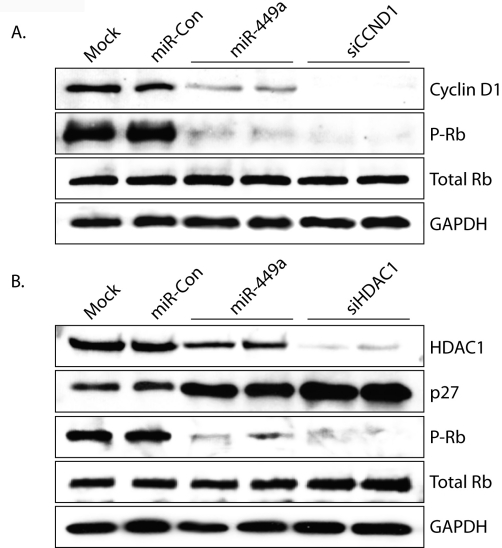
miR-449a regulates Rb phosphorylation by targeting Cyclin D1 and HDAC1 **A.** PC-3 cells were transfected at 50 nM concentrations of miR-Con, miR-449a, or siCCND1 for 72 hours. Mock samples were transfected in the absence of miRNA/siRNA. Cyclin D1, phosphorylated Rb (P-Rb), total Rb, and GAPDH protein levels were evaluated by immunoblot analysis using protein-specific antibodies. **B.** PC-3 cells were transfected with mock, miR-Con, miR-449a, or siHDAC1 for 72 hours as indicated. HDAC1, p27, P-Rb, total Rb, and GAPDH protein levels were evaluated by immunoblot analysis. GAPDH served as a loading control.

Hyperphosphorylation of Rb promotes cell cycle progression and cell growth [26, 27]. To determine if miR-449a regulates Rb activity, we evaluated Rb phosphorylation by immunoblot analysis following knockdown of Cyclin D1. We transfected PC-3 cells with miR-449a or a specific siRNA designed to target only Cyclin D1 (siCCND1). As shown in Figure 6A, knockdown of Cyclin D1 by miR-449a or siCCND1 drastically reduced phophorylated Rb (P-Rb) levels. This data indicates that miR-449a regulates Rb phosphorylation, in part, through targeted knockdown of Cyclin D1.

Cell cycle inhibitory protein p27 also functions to regulate Rb phosphorylation by inhibiting cyclin dependent kinase (CDK) activity [28]. It has previously been shown that miR-449a activates p27 expression by targeted knockdown of HDAC1 in prostate cancer cells [16]. To determine if miR-449a can also modulate Rb activity through HDAC1, we transfected PC-3 cells with miR-449a or a specific siRNA targeting HDAC1 (siHDAC1) and detected Rb phosphorylation by immunoblot analysis. As shown in Figure 6B, knockdown of HDAC1 by miR-449a or siHDAC1 elevated p27 protein and reduced P-Rb levels. This data indicates that miR-449a also regulates Rb phosphorylation though knockdown of HDAC1.

## DISCUSSION

**Figure 7:**
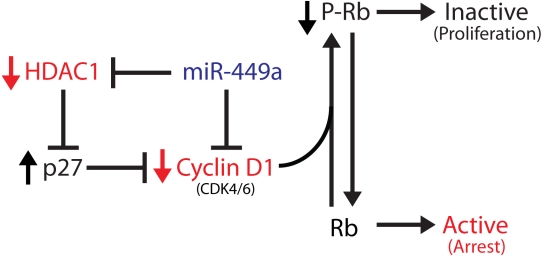
A model linking miR-449a to Rb activation and growth arrest in prostate cancer cells Direct downstream targets of miR-449a are shown in red, while miR-449a is colored blue. miR-449a-mediated depletion of Cyclin D1 and/or HDAC1 reduces Rb phosphorylation leading to growth arrest (denoted in red type).

We provide evidence that the putative tumor suppressor function of miR-449a is, in part, dependent on Rb in prostate cancer cells. DU-145 cells devoid of wild-type Rb are resistant to cell cycle arrest and senescence induced by miR-449a. Only upon restoration of Rb did miR-449a regain its growth inhibitory effects in the DU-145 sublines. Based on our data, a simple model can be created linking miR-449a to Rb activation and growth arrest in prostate cancer cells (Figure 7). We show that miR-449a can regulate Rb phosphorylation by directly targeting Cyclin D1 and HDAC1. Modulation of Rb activity through HDAC1 knockdown is likely facilitated through downstream targets such as the activation of p27 expression. Interestingly, p27 directly binds cyclins (e.g. Cyclin D1) and inhibits CDK4/6 complex activity [28]. By this mechanism, miR-449a has a dual approach for regulating Cyclin D1 activity and Rb phosphorylation.

It has also been reported that miR-449a directly targets and represses the expression of CDK6 and CDC25A; two more key factors involved in promoting Rb phosphorylation [29]. Further evidence has indicated that miR-449a is also transcriptionally regulated by E2F transcription factors [29-31]. E2F proteins preferentially interact with hypophosphorylated Rb, which sequesters their activity. Upon Rb phosphorylation, E2F proteins are released to activate downstream gene expression. Because E2F family members function as downstream mediators of Rb, miR-449a functions in an auto-regulatory loop to control Rb activity by targeting multiple upstream regulatory factors (i.e. Cyclin D1, HDAC1, CDK6, and CDC25B). Collectively, these reports, in combination with our data, define miR-449a as an integral miRNA component of the Rb pathway.

Phenotypically, miR-449a is a multifaceted miRNA in that it induces apoptosis in association to cell cycle arrest [16, 30]. However, we reveal that miR-449a promotes apoptosis in prostate cancer cells regardless of Rb status; both PC-3 and DU-145 cells are susceptible to the apoptotic effects of miR-449a. Previous research has indicated that the specific knockdown of HDAC1 contributes to the apoptotic effects of miR-449a [16]. Because HDACs regulate numerous downstream factors including apoptotic genes (i.e. Bcl-XL, etc.), miR-449a may promote apoptosis in an Rb-independent manner through depletion of HDAC1 [32, 33]. In addition, miR-449a may also directly target and suppress the expression of antiapoptotic genes. For instance, *in silico* analysis utilizing the miRanda algorithm reveals a putative target site in the 3'UTR of the BCL2 transcript [25]. We also reveal that wild-type Rb enhanced the apoptotic response in DU-145 sublines to suggest that miR-449a may facilitate apoptosis through Rb-dependent mechanisms, as well. In support, Rb activation is known to trigger apoptosis by sequestering E2F protein activity and repressing the expression of downstream antiapoptotic gene BIRC5/survivin [34].

Precise control of Rb activity is absolutely essential for maintaining regulated cellular growth. In nearly all cancer types Rb is inactivated to promote oncogenesis. We propose that the loss of miR-449a expression can promote Rb inactivation and prostate cancer progression. *In vivo* analysis has shown that miR-449a is depleted in human prostate tumor tissue relative to patient-matched controls [16]. Furthermore, miR-449a is located in a chromosomal region previously identified as a susceptibility locus in a variety of malignancies including prostate cancer [35, 36]. The mechanism by which miR-449a is depleted in prostate cancer may result from genomic deletion or epigenetic silencing. Co-treatment of histone methylation and HDAC inhibitors has been shown to re-activate miR-449a expression in breast cancer cells [29]. Regardless, loss of miR-449a would disrupt its auto-regulatory control over Rb and promote unregulated growth, which may, in part, contribute to transformation during prostate cancer tumorigenesis. Our data supports the tumor suppressor-like function of miR-449a by highlighting its relationship with Rb and describing its inhibitory effects on cell growth. Although miR-449a-mediated cell cycle arrest is largely Rb-dependent, re-activation or replacement of miR-449a may have therapeutic benefit in prostate cancer that retains functional Rb status.

## MATERIAL AND METHODS

### Cell culture and miRNA/siRNA transfection

PC-3, DU-145, DuPro, DU-1.1, and B5 cells were maintained in RPMI 1640 medium supplemented with 10% FBS, L-glutamine (2 mM), penicillin (100 U/ml) and streptomycin (100 μg/ml) in a humidified atmosphere of 5% CO_2_ at 37°C. DU-1.1 and B5 are well characterized DU-145 sublines engineered to express wild-type Rb protein [23, 37]. The day before transfection, cells were plated in growth medium without antibiotics at a density of ~50-60%. Transfection of miRNA/siRNA was carried out using Lipofectamine RNAiMax (Invitrogen, Carlsbad, CA) according to the manufacturer's instructions for 72 hours. The mature hsa-miR-449a mimic (miR-449a), non-specific control (miR-Con), Cyclin D1 (siCCND1) and HDAC1 (siHDAC1) siRNAs were synthesized by Invitrogen. All duplexes contained 2-nucleotide 3' overhangs. Sequences are listed in Supplementary Table 1.

### Analysis of cell cycle distribution and apoptosis by flow cytometry

Transfected cells were trypsinized and centrifuged at 2000×g for 5 min at 4°C in complete medium. Cell pellets were resuspended in 1 ml of cold saline GM solution (6.1 mM glucose, 1.5 mM NaCl, 5.4 mM KCl, 1.5 mM Na2HPO_4_, 0.9 mM KH2PO4, 0.5 mM EDTA) and fixed in 3 ml of 100% ethanol overnight at 4°C. Cells were then washed once in PBS containing 5 mM EDTA, centrifuged at 2000×g for 5 min, and resuspended in 1 ml of propidium iodide (PI) staining solution (30 μg/ml PI, 300 μg/ml RNase A in PBS). Cells were stained for 1 hour at room temperature in the dark and subsequently filtered through 30 μm nylon mesh. Analysis was performed on a FACSCalibur flow cytometer (Becton Dickinson, Franklin Lakes, NJ). A total of 10,000 events were collected and PI intensity was analyzed using the FL2 channel for relative DNA content. Forward and side scatter gates and a doublet discrimination plot were set to include whole and individual cell populations, respectively. The resulting data was analyzed to determine cell cycle distribution and sub-diploid/apoptotic cell fraction. Markers were placed to quantify the percentage of cells in sub-diploid, G0/G1, S, and G2/M populations.

### Senescence-associated β-galactosidase (SA-β-gal) staining

PC-3, DU-145, DuPro, DU-1.1, and B5 cell lines were transfected with miR-449a for 72 hours and stained for SA-β-gal activity as previously described [21]. Briefly, cells were washed with PBS, fixed in 3% formalhehyde for 10 min at room temperature, and incubated overnight at 37°C in SA-β-gal staining reagent (1 mg/ml of X-Gal, 5 mM potassium ferrocyanide, 5 mM potassium ferricyanide, 150 mM NaCl, and 2 mM MgCl2, 40 mM citric acid/sodium phosphate, pH 6.0). Cell images were taken at 200× magnification by phase contrast microscopy.

### Immunoblotting

Cultured cells were washed with cold PBS and lysed with M-PER protein extraction buffer (Pierce, Rockford, IL) containing protease inhibitors. Cell lysates were centrifuged and supernatants were collected. Equal quantities of protein were resolved by electophoresis on sodium dodecyl sulfate (SDS) polyacrlamide gels and transferred to 0.45 μm nitrocellulose membranes by voltage gradient. The resulting blots were blocked with 5% non-fat dry milk and probed with primary antibodies specific to Rb (Cell Signaling, Danvers, MA), P-Rb (BD Transduction, Franklin Lakes, NJ), HDAC1 (Santa Cruz Biotechnology, Santa Cruz, CA), p27 (BD Transduction), or GAPDH (Chemicon, Temecula, CA). Immunodetection occurred by incubating blots with appropriate secondary HRP-linked antibodies and utilizing chemiluminescence to visualize the antigen-antibody complexes. GAPDH served as an internal control.

### 3'UTR constructs/luciferase assay

Complementary oligonucleotides containing the putative miR-449a target site from Cyclin D1 (CCND1-WT) was cloned into the 3'UTR of the pMIR-Report luciferase reporter vector (Applied Biosystems, Foster City, CA). A scrambled target site (CCND1-MUT) designed to interfere with seed sequence recognition was also cloned to serve as a control for specificity. All oligonucleotide sequences used to create the 3'UTR constructs are listed in Supplementary Table 1. PC-3 cells were transfected with 0.6 μg CCND1-WT or CCND1-MUT construct, 0.4 μg pMIR-Report Beta-gal, and 30 nM miR-449a for 24 hours. The pMIR-Report Beta-gal vector served as a control to monitor transfection efficiency. Treatment with miR-449a anti-miR inhibitory oligonucleotide (anti-miR-449a) or anti-miR negative control (anti-miR-Con) from Applied Biosystems was also utilized to sequester miR-449a activity and validate sequence specificity. The Dual-Light System® chemiluminescent reporter gene assay (Applied Biosystems) was used to quantify luciferase and β-galactosidase activity.

## SUPPLEMENTARY MATERIAL



## Abbrevation Used

3'UTR: 3' untranslated region
HDACs: histone deacetylases
