# Supplementary material for: miR-449a causes Rb-dependent cell cycle arrest and senescence in prostate cancer cells
Source: Oncotarget. 2010 Sep 13;1(5):349–58. doi: 10.18632/oncotarget.167 (PMC2952964; doi:10.18632/oncotarget.167)
Supplement: Supplementary file 1 [file oncotarget-01-349-s001.doc]

**miR-449a causes Rb-dependent cell cycle arrest and senescence in prostate cancer cells**

Emily J. Noonan, Robert F. Place, Shashwati Basak, Deepa Pookot, and Long-Cheng Li

**SUPPLEMENTARY INFORMATION**


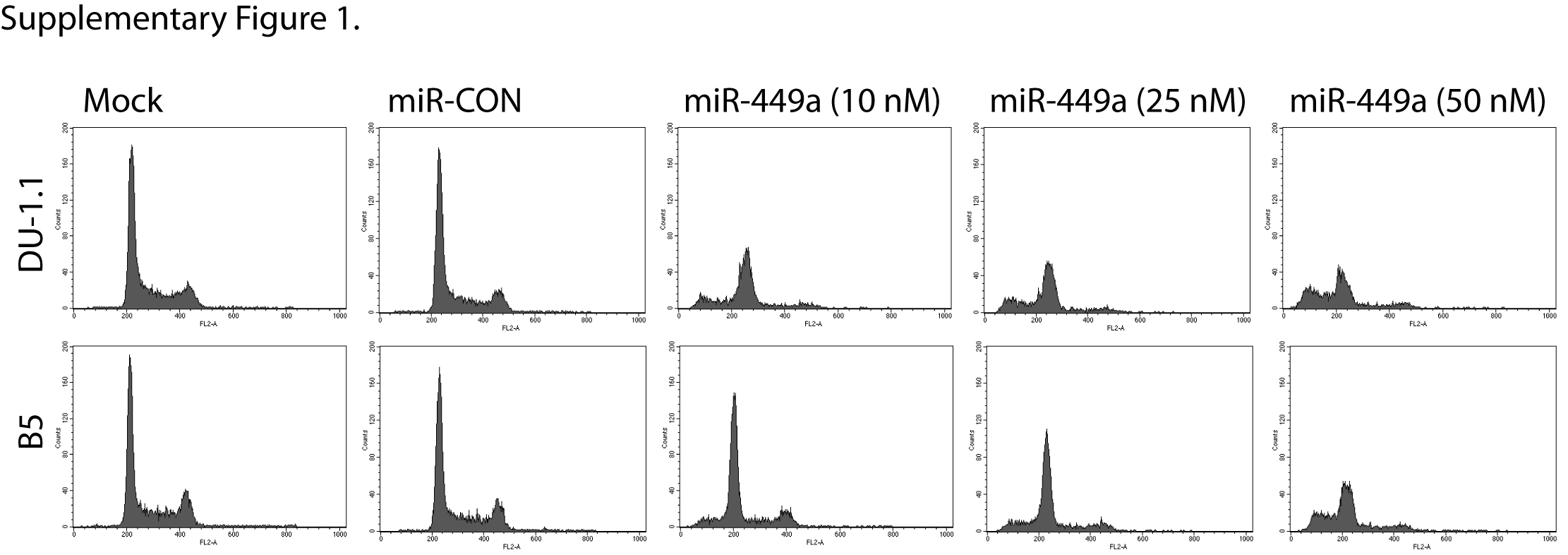


**Supplementary Figure 1. Representative histograms of DU-1.1 and Rb5 cell cycle distribution.** DU-1.1 and Rb5 cells were transfected at different concentrations of miR-449a for 72 hours as indicated. Floating and attached cells were collected, stained with PI, and processed for analysis by flow cytometry to measure DNA content. Shown are examples of resulting FL2A histograms.


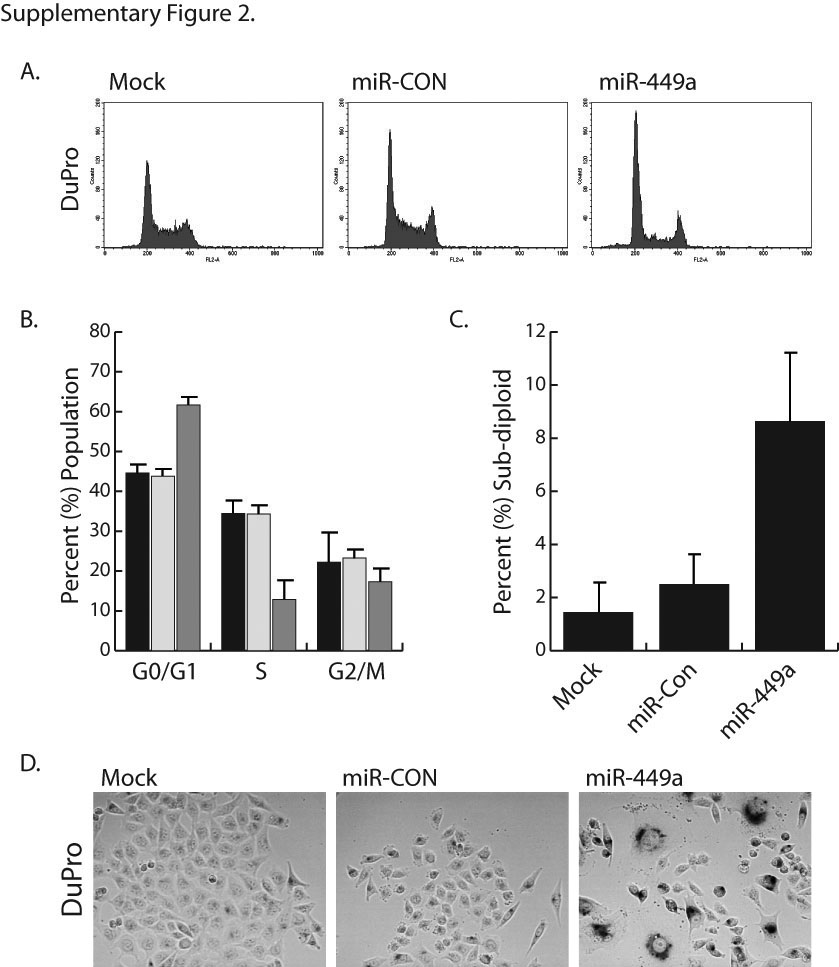


**Supplementary Figure 2. DuPro cells are sensitize to growth arrest and senescence induced by miR-449a. A.** DuPro cells were transfected with 50 nM concentrations of miR-CON or miR-449a for 72 hours. Mock samples were transfected in the absence of miRNA. Floating and attached cells were collected, stained with PI, and processed for analysis by flow cytometry to measure DNA content. Shown are examples of resulting FL2A histograms. **B.** Flow cytometry data was analyzed to determine cell cycle distribution (G0/G1, S, and G2/M) in the surviving cell populations. **C.** Percentages of sub-diploid/apoptotic cells were calculated from entire gated whole-cell populations. **D.** DuPro cells were transfected with mock, miR-CON, or miR-449a for 72 hours. Cells were fixed in formaldehyde and stained for SA-β-gal activity overnight. Images were captured by phase contrast microscopy at 200X magnification. Dark perinuclear staining marks senescent cells.

**
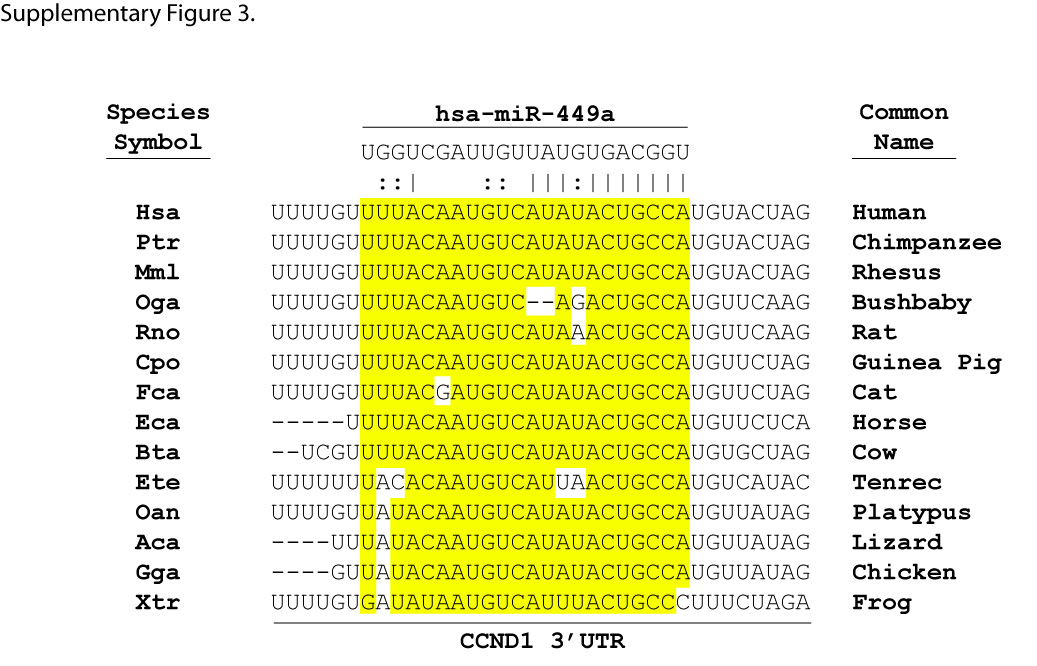
**

**Supplementary Figure 3. Sequence conservation of the miR-449a target site in the CCND1 3’UTR.** Indicated is complementary base-pairing, including G:U wobbles, between human mature miR-449a (hsa-miR-449a) and its target site in the 3’UTR of the Cyclin D1 (CCND1) transcript. Sequence alignment and conservation across the indicated species was performed using the TargetScan search engine ([http://www.targetscan.org](http://www.targetscan.org/)). Conserved nucleotides are highlighted in yellow. Species symbols and common names are also shown.

**Supplementary Table 1.** miRNA/siRNA and oligonucleotide sequences.
